# MiRNA-182-5p aggravates experimental ulcerative colitis via sponging Claudin-2

**DOI:** 10.1007/s10735-021-10021-1

**Published:** 2021-10-08

**Authors:** Siwen Tang, Wentao Guo, Liumin Kang, Jinghua Liang

**Affiliations:** 1 Shenzhen Traditional Chinese Medicine Anorectal Hospital (FuTian), No. 1 Songling Road, Futian District, 518000 Shenzhen, China; 2 Suzhou Science and Technology City Hospital Affiliated to Nanjing Medical University, 215000 Suzhou, China

**Keywords:** MiRNA-182-5p, Claudin-2, Colitis, Inflammation, Intestinal mucosal barrier, Anti-oxidative

## Abstract

Tight junction proteins play crucial roles in maintaining the integrity of intestinal mucosal barrier. MiRNA-182-5p is capable of targeting claudin-2 which is one of the vital tight junction proteins and the effect and mechanism of miRNA-182-5p was explored here in the DSS-induced colitis model. The pathological conditions were evaluated via hematoxylin and eosin staining. The gene expression level was assessed via PCR. Quantitative immunohistochemistry analysis was performed for the measurement of claudin-2. microRNA.org online tool was used for target gene prediction. Luciferase reporter assay and RNA pull-down assay were performed to detect the target of miRNA-182-5p. The inflammatory and oxidative stress level were measured using corresponding kits. MiRNA-182-5p was highly expressed in colitis model and miRNA-182-5p inhibitor exerted protective effects on colitis induced by DSS in mice. The protective effects includded improvement of pathological changes, increases in anti-inflammation and anti-oxidative genes, and up-regulation of TGF-β1. Claudin-2 mRNA was predicted as the target of miRNA-182-5p, which was validated via luciferase reporter assay and RNA pull-down assay. Claudin-2 overexpression was found in miRNA-182-5p inhibitor group. Consistent with the role of miRNA-182-5p, claudin-2 overexpression also exerted protective effects on DSS-induced colitis in mice. Inhibition of miRNA-182-5p exerted protective effects on colitis via targeting and upregulating claudin-2. The findings in study provide a new therapeutic strategy for colitis treatment and lay the foundation for future study.

## Introduction

Inflammatory bowel disease is a refractory, nonspecific, debilitating inflammatory disease [1]. Ulcerative colitis and Crohn’s disease are two major forms of inflammatory bowel disease [2]. Common clinical manifestations include abdominal pain, fever, diarrhea, hematochezia, tenesmus, vomiting, and an increase in frequency of defecation [3, 4]. The incidence of inflammatory bowel disease is soaring globally [5–7]. In addition, chronic colorectal inflammation has been confirmed as the vital contributor of tumorigenesis [8]. Nowadays, the known causes of inflammatory bowel disease includes environmental factors, genetic factors and immunity factors [9]. The exact pathogenesis is complex and yet to be further elaborated. The current treatment such as immunomodulators and antibiotic can only achieve clinical remission [10]. Meanwhile, there is individual difference in patients with inflammatory bowel disease, which has made the treatment more difficult. Therefore, it’s urgent to elaborate the vital regulatory mechanism without individual difference and develop effective therapeutic drugs based on it.

MicroRNAs (miRNAs), as non-coding RNAs, function by inhibiting the translation or promoting the degradation of mRNA, which are powerful gene regulators of gene expression at transcriptional and post-transcriptional levels. miRNAs have been involved in a lot of cell activities including metabolism, cell proliferation and differentiation, cell cycle, aging, immune responses, inflammation and so forth, which are also served as the vital biomarkers and therapeutic targets in many diseases [12–15]. MiRNA-219a-5p is found to have inhibitory effects on intestinal inflammation via inhibition of immune responses mediated by T helper (Th)1/Th17, which may become a promising therapeutic target for colitis [16]. MiRNA-125a inhibits the inflammation of intestinal mucosal via targeting ETS-1 in patients [17]. Therefore, it is beneficial to screen for? the vital miRNA that is crucial in the onset and progress of colitis.

Increasing evidence has shown that intestinal mucosal barrier defects are the core factors for IBD [18]. When the intestinal mucosal barrier is impaired, the intestinal lamina propria will be invaded by toxins and pathogenic bacteria [19, 20]. The intestinal mucosal barrier is mainly maintained by the tight junction proteins which consist of claudins, zonula occluden-1 and occluding. Claudin-2 as one of the tight junction proteins is downregulated in inflammatory bowel disease, which contributes to the further progress of IBD [21]. Reducing the decrease of Claudin-2 may be a good therapeutic strategy for IBD. It is promising to find the vital miRNA that is capable of regulating Claudin-2 expression. After bioinformatics analysis (microRNA.org online tool), Claudin-2 is predicted to be the target gene of miRNA-182-5p and the effects of miRNA-182-5p on inflammatory bowel disease remains unknown. Herein, we are the first to investigate the effects of miRNA-182-5p and its association with Claudin-2 in dextran sulphate sodium (DSS) induced colitis.

## Materials and methods

### Experimental animals

The C57BL/6 mice (7–8 week, n = 80) from Cancer prevention and treatment center of Sun Yat sen University (SYXK(yue)2021 − 0255) were raised under specific pathogen free conditions with humidity of 65% at 22 °C in the experimental animal center. This study is approved by committee of Shenzhen Traditional Chinese Medicine Anorectal Hospital and the whole animal experimental processes were performed in line with the Committee guidelines of animal research.

### The establishment of experimental colitis model and treatment [22]

After getting acclimatized to the environment, the mice (ten mice per group) were randomly divided into control group, model group, negative control (NC) group and inhibitor group.

The mice in the model group were treated with 4% DSS (MP Biomedicals, Santa Ana, California, USA) for one week. The mice in control group were treated with equal volume of sterilized water. For NC group, the mice after establishment of experimental colitis were treated with miRNA-182-5p negative control (200 µl, Sangon Biotech (Shanghai) Co., Ltd. Shanghai, China) via intravenous injection. For inhibitor group, DSS mice were treated with miRNA-182-5p (200 µl, Sangon Biotech) via intravenous injection. For Claudin-2 NC group, the mice after establishment of experimental colitis were treated with negative control of plasmid (200 µl, Sangon Biotech) via intravenous injection. For Claudin-2 group, the mice after establishment of experimental colitis were treated with Claudin-2 overexpression plasmid (200 µl, Sangon Biotech) via intravenous injection. During the period, the stool consistency, body weight and intestinal bleeding were monitored every day.

### Hematoxylin and eosin staining

After treatment, the mice in the study groups were sacrificed via euthanasia. The colonic tissues were dissected, isolated and washed with cold PBS. Then the samples were fixed with 4% paraformaldehyde. Embedded in paraffin, the samples were sliced into 5 μm sections. Then the deparaffinization was performed using xylene and the samples were rehydrated with gradient elution. Subsequently, the hematoxylin was added to stain the sections for ten minutes and eosin for one minute. After dehydration, the sections were vitrificated and examined using microscope (Olympus Corporation, Japan).

### Immunohistochemical analysis

After treatment, the mice in the study groups were sacrificed via euthanasia. The colonic tissues were dissected, isolated and washed with cold PBS. Then the samples were fixed with 4% paraformaldehyde. Embedded in paraffin, the samples were sliced into 5 μm sections. The sections were heated in the PBS in a microwave. After cooling down, the serum blocking solution was added to block the sections for half an hour at 37 °C. Then the sections were incubated with anti-Claudin-2 antibody(1:1000, #ab53032, Abcam, England) at 4 °C overnight and secondary antibody (#ab205718, Abcam) at 37 °C for one hour. After being washed with PBS, the sections were stained with diaminobenzidine for five minutes and hematoxylin for two minutes. Finally, the sections were observed under the microscope (Olympus Corporation).

### Real-time qRT-PCR assay (quantitative real-time PCR qRT-PCR)

TRIzol reagent (Thermo Fisher Scientifc, Inc., Waltham, MA, USA.) was used for istolating total RNAs from colonic tissues according to the protocol of manufacturer. MiScript II RT Kit (218161, Qiagen, Germany) was used for the reverse transcription from miRNA to cDNA. The PCR assay were performed on RT-qPCR detection system (Applied Biosystems; Thermo Fisher Scientific, Inc., U.S.A.). The amplification conditions and miR-182-5p primers were the same with the previous study [23]. The primers were obtained from Sangon Biotech for Claudin-2 F:5′-TTCATCGGCAACAGCATCG-3′ (Forward) and R:5′-GGTTATAGAAGTCCCGGATGA-3′ (reverse);

### Assessment of colonic inflammation and damage

The colonic tissues were homogenized and submitted for centrifugation (20000× g, 25 min). The supernatants were collected for detection. The inflammatory factors including transforming growth factor (TGF)-β, interleukin (IL)-21, IL-17, IL-6 in the supernatants were assessed herein via using the corresponding ELISA kits (eBioscience) according to the manufacturer’s protocols, including TGF-β1(Transforming Growth Factor Beta 1) ELISA Kit (Cat: E-EL-0162c; Elabscience Biotechnology Co., Ltd, Wuhan, China), Mouse Interleukin-21 ELISA Kit (Cat: ZN2661; Beijing baiaolaibo Technology Co., Ltd, Beijing, China), Mouse Interleukin 17,IL-17 ELISA Kit (Cat: E-EL-M0047c; Elabscience Biotechnology) and Mouse IL-6(Interleukin 6) ELISA Kit (Cat: E-EL-M0044c; Elabscience Biotechnology). The colonic damage index was evaluated according to the previous report [24]. The colonic damage index was scored on a scale of 0–5. The entire colon of each animal was rinsed with cold physiological saline to remove feces. The colon damage was examined visually and immediately. The colonic damage was scored as 0, for no damage; 1, for hyperemia and no ulcers; 2, for linear ulcer with no significant inflammation; 3, for Linear ulcer with inflammation at one site; 4, for two or more sites of ulceration or inflammation and the area extending < 1 cm; 5, for two or more major sites of ulceration or inflammation extending > 1 cm.

### Assessment of MDA and SOD level

After treatment, the mice in the study groups were sacrificed via euthanasia. The colonic tissues were dissected. The malondialdehyde (MDA) in the supernatants were detected as described before [25]. The superoxide dismutase (SOD) level in the supernatants was detected using Superoxide Dismutase Activity Assay Kit (Cat: E-BC-K020-M; Elabscience Biotechnology). The detection of SOD was performed according to the manufacturer’s protocols.

## Luciferase reporter assay [26]

Claudin-2 is predicted as the target of miRNA-182-5p through microRNA.org online tool (http://microRNA.org). The 3′UTR sequence of claudin-2 (WT) or its mutated type (MUT) was cloned into the psiCHECK2 vector (Invitrogen). The miR-182-5p or miR-182-5p mimic with mutant or wild-type of claudin-2 were co-transfected into HEK293 cells using Lipofectamine 2000 (Thermo Fisher Scientifc) in accordance with the protocols of manufacturer. After transfection for 48 h, luciferase activity was detected via using dual luciferase reporter assay system (Promega Corporation, Madison, USA).

### RNA pull-down assay [27]

The normal intestinal epithelial cell line Caco-2 was purchased from iCell Biotechnology Co., Ltd (Shanghai, China). The cells were cultured in RPBM 1640 medium supplemented with 10 % fetal calf serum and maintained at 37 ℃ with 5% CO_2_ in a humidified atmosphere. The RNA pull-down assay was performed via using Magna RIP Kit (Millipore (China) Co., Ltd, Shanghai, China). The cells were transfected with bio-wild-miRNA-182-5p (miRNA-bio), bio-mutated miRNA-182-5p (miRNA-MUT-bio) or NC-bio through Lipofectamine reagent following the manufacturer’s recommendations (Thermo Fisher Scientifc). 48 h after transfection, the cells were lysed to collect the lysates in different study group. Then the lysates were incubated with streptavidin magnetic beads. Finally. The PCR was performed for detection of the immunoprecipitated RNA.

### Statistical analysis

The results from three individual experiments are analyzed via using SPSS 20.0 software (IBM, CA, USA). The final data were presented as mean ± standard deviation. The difference comparation was performed via using one-way ANOVA. *P < 0.05 represents statistical significance.

## Results

**Fig. 1:**
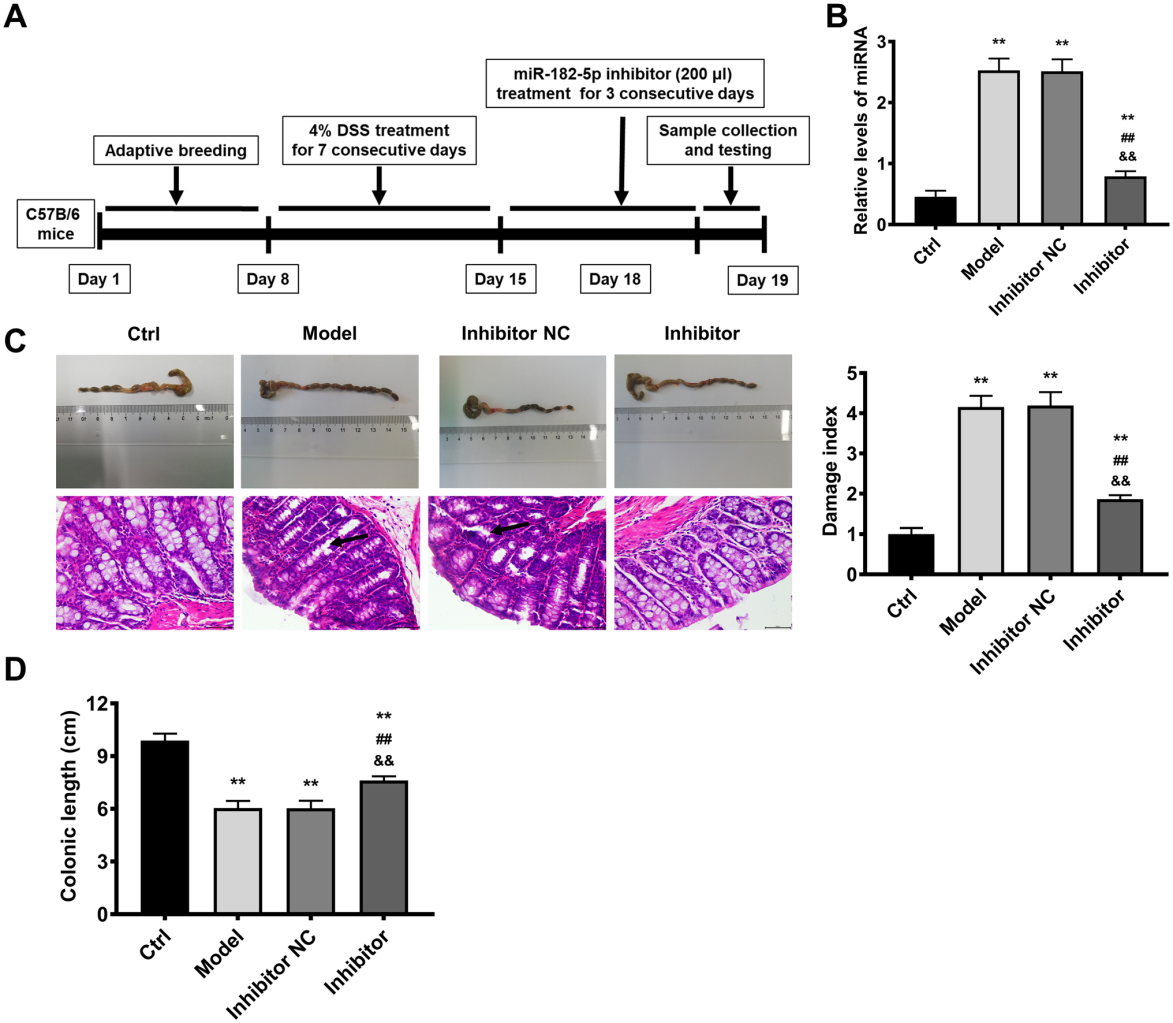
Downregulation of miRNA-182-5p exerts protective effects on colitis stimulated by DSS in mice. The timeline of miRNA-182-5p effects on colitis stimulated by DSS in mice (**A**); The miRNA-182-5p level evaluated by PCR in the different study groups (**B**); The colonic length, representative images of H&E staining and damage index in the study groups, the scales = 50 μm (**C** and **D**) **P < 0.01 Vs. Control group; ^##^P < 0.01Vs. model group; ^&&^P < 0.01 Vs. inhibitor NC group

MiRNA-182-5p deficiency exerted protective effects on colitis induced by DSS in mice. Firstly, we established the experimental colitis model (Fig. 1A). Compared with control group, the miRNA-182-5p level was up-regulated in the model group (Fig. 1B), suggesting that the aberrant expression of miRNA-182-5p may be a potential therapeutic target in colitis. Therefore, we further evaluated the role of silencing miRNA-182-5p in colitis. After miRNA-182-5p inhibitor treatment, the miRNA-182-5p level in colitis was downregulated significantly. Compared with control group, the colon length was shortened significantly, indicating that the colon was severely damaged (Fig. 1C). In the mean time, as shown by the H&E staining results, the histological structure was normal and there was no sign of inflammatory infiltration in control group (Fig. 1C). In contrast, submucosal edema, goblet cells damage and inflammation infiltration were observed in the colitis model group. In addition, the colonic damage index was elevated evidently in model group (Fig. 1D). All these results proved that the colitis model was established successfully. Moreover, the histopathological changes, shortening of colonic length and colonic damage index were all reduced by miRNA-182-5p inhibitor, confirming that miRNA-182-5p deficiency have protective effects on colitis induced by DSS.

**Fig. 2:**
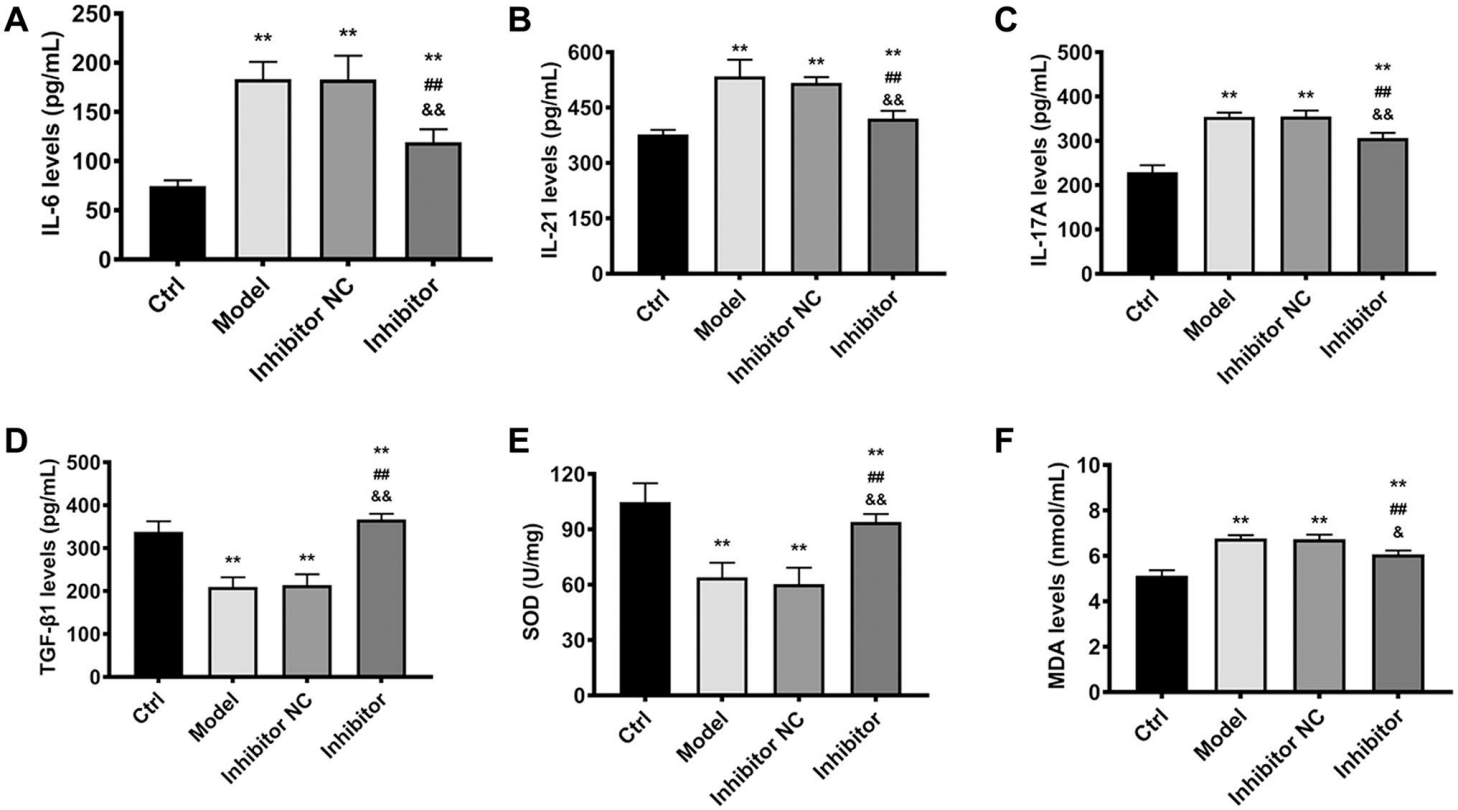
The effects of miRNA-182-5p on inflammatory factors, oxidative stress and TGF-β1 level. The inflammatory factors, oxidative stress and TGF-β1 level in the study groups. **P < 0.01 Vs. Control group; ^##^P < 0.01Vs. model group; ^&&^P < 0.01 Vs. inhibitor NC group

Inhibition of miRNA-182-5p exerted protective effects in colitis model via suppression of inflammation response as well as oxidative stress and elevation of TGF-β1. The inflammatory factors IL-21, IL-17 A, IL-6 were up-regulated significantly in colitis model group in contrast to control, while miRNA-182-5p inhibitor alleviated the increase of IL-21, IL-17 A, and IL-6 (Fig. 2A–C). TGF-β1 is a crucial wound healing factor and has promotional effect on tight junction protein expression [28]. TGF-β1 was decreased remarkably in colitis group when compared with control. MiRNA-182-5p inhibitor treatment resulted in significant increase in TGF-β1 level as compared with model group (Fig. 2D). SOD and MDA as indicators of oxidative stress were also evaluated herein. Compared with control, the SOD was declined and MDA was increased obviously in colitis model group, suggesting that the oxidative stress was enhanced in model group, while inhibitor alleviated the decrease of SOD and the increase of MDA (Fig. 2E, F). The findings herein confirmed that miRNA-182-5p could alleviate colonic damage via elevation of TGF-β1 and suppression of inflammation response and oxidative stress.

**Fig. 3:**
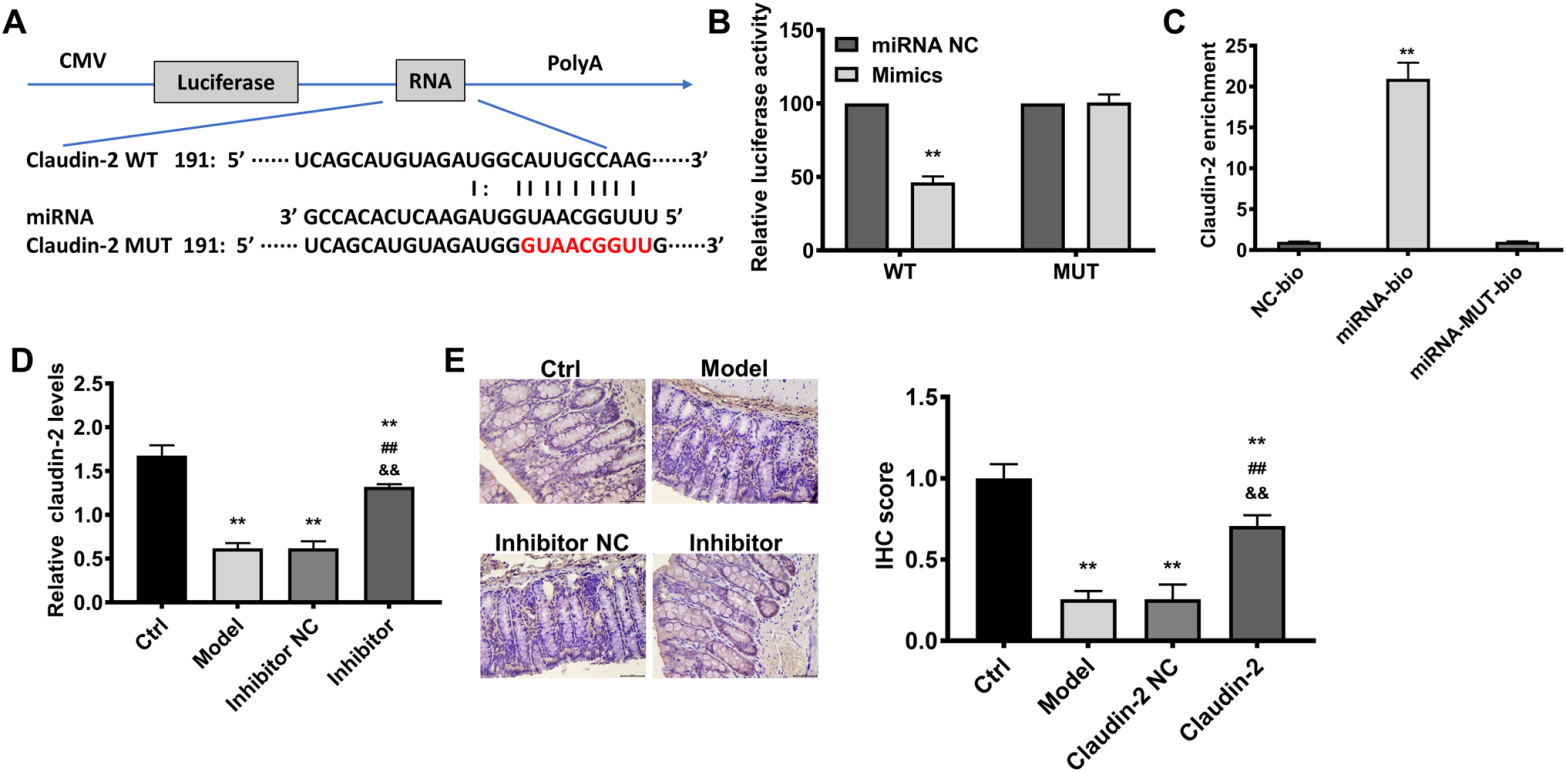
Predication and validation of the target of miRNA-182-5p. The target binding sites predicted via microRNA.org online tool (A) and validated via luciferase reporter assay and RNA pull-down assay (B and C); The Claudin-2 levelsin the study groups, the scales= 50 μm(D and E). **P<0.01 Vs. Control group; ##P<0.01Vs. model group; &&P<0.01 Vs. inhibitor NC group

Claudin-2 was the binding target of miRNA-182-5p and was negatively regulated by miRNA-182-5p level in colitis model. Claudin-2 was predicted to be the target of miRNA-182-5p via online tool (microRNA.org) (Fig. 3A). This was further validated via luciferase reporter assay and RNA pull-down assay (Fig. 3B, C). The lowest luciferase activity and claudin-2 enrichment level was found in (WT) RNA mimic group and miRNA-bio group respectively, strongly supporting that claudin-2 was targeted by miRNA-182-5p. The claudin-2 level was declined evidently in colitis group. As shown by PCR and immunohistochemical results, miRNA-182-5p inhibitor caused a significantly increase in claudin-2 level as compared with control (Fig. 3D, E), suggesting that the claudin-2 level was negatively regulated by miRNA-182-5p.

**Fig. 4:**
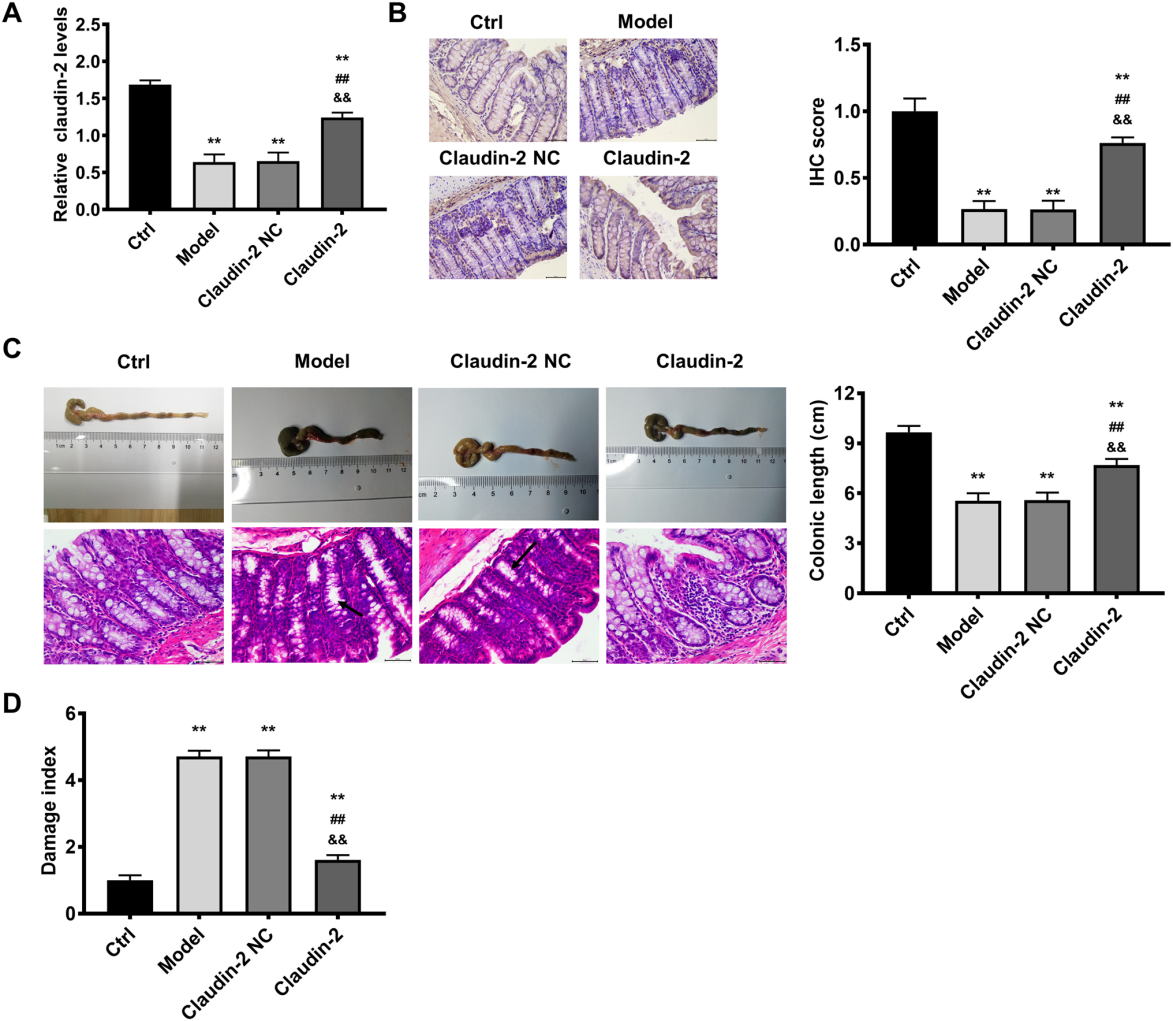
Claudin-2 overexpression exerts protective effects on colitis induced by DSS in mice. Claudin-2 levels in the study groups (**A**, **B**); The colonic length, representative images of H&E staining and damage index in the study groups, the scales = 50 μm (C and D). **P < 0.01 Vs. Control group; ^##^P < 0.01Vs. model group; &&P < 0.01 Vs. Claudin-2 NC group

**Fig. 5:**
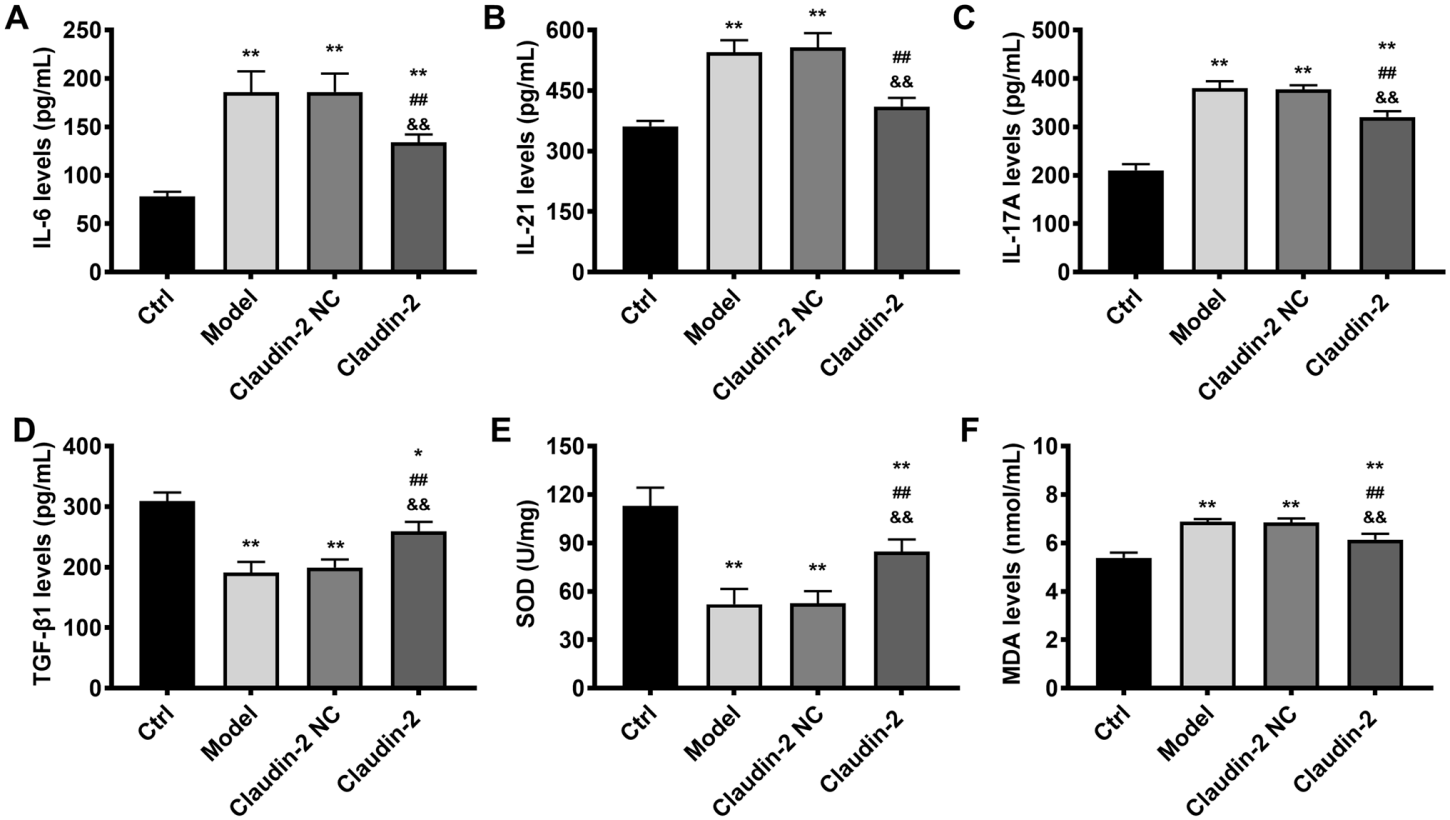
The effects of claudin-2 overexpression on inflammatory factors, oxidative stress and TGF-β1 level. The inflammatory factors, oxidative stress and TGF-β1 level in the study groups. **P < 0.01 Vs. Control group; ^##^P < 0.01Vs. model group; ^&&^P < 0.01 Vs. Claudin-2 NC group

Silencing miRNA-182-5p alleviated colonic damage via targeting claudin-2. As claudin-2 was confirmed as the target of miRNA-182-5p, the following experiments was performed to determine whether miRNA-182-5p functions via targeting claudin-2. If miRNA-182-5p functions via targeting claudin-2, the effects of miRNA-182-5p inhibitor will be in accordance with claudin-2 overexpression. Consistent with the results above, claudin-2 was reduced significantly in colitis model and overexpressed evidently in claudin-2 overexpression group (Fig. 4A, B). The colonic length, histopathological image and colonic damage index (Fig. 4C, D ) in control or model group were consistent with the results in Fig. 1 as described above. Claudin-2 overexpression resulted in improvement in the shortening of colonic length, the histopathological changes and colonic damage index, which was consistent with the effects of miRNA-182-5p inhibitor in colitis model. The effects of claudin-2 overexpression on inflammatory factors, oxidative stress and TGF-β1 were also in line with the effects of miRNA-182-5p inhibitor (Fig. 5). The increase of IL-6, IL-21, IL-17 A and MDA were partially inhibited by claudin-2 overexpression. While the decrease of TGF-β1 and SOD were partially inhibited by claudin-2 overexpression. All the findings suggested that silencing miRNA-182-5p protected against colonic injuries via targeting claudin-2.

## Discussion

Inflammatory bowel disease is one of the major chronic digestive system diseases with features of increasing incidence and threatening patients’ quality of live [7]. The gut epithelium is the crucial barrier against pathogen invasion. The epithelial barrier impairment has been confirmed as the main etiological factor for colitis [29–32]. Increasing studies have showed that miRNAs have played a vital role in the regulation of epithelial barrier function. Intestinal epithelial barrier function can be enhanced via miR-381-3p knockdown [33]. MiR-24 is reported to act as a crucial regulator in intestinal barrier [34]. MiR-429 overexpression can cause intestinal barrier dysfunction [35]. Therefore, it’s meaningful to find the aberrantly expressed miRNA which acts as a vital regulator in the onset and progress of colitis.

Firstly, we established the colitis model via using DSS. Consistent with colitis model in the literatures, the colonic length was shortened evidently and the damage index was elevated [36–38]. In addition, as seen from the H&E staining, submucosal edema, goblet cells damage and inflammation infiltration occurred in the colitis model which is also consistent with previous reports [37, 38].

MiRNA-182-5p is a new emerging miRNA and has been confirmed to be involved in several diseases [23, 39–42]. In this study, miRNA-182-5p was found to be up-regulated significantly in colitis model group, which indicates an underlying role of it in colitis. We speculate that inhibition of miRNA-182-5p may help to alleviate ulcerative colitis We conjectured that inhibition of miRNA-182-5p will alleviate experimental ulcerative colitis. Just as expected, the colonic length, damage index and pathological lesion were all improved in miRNA-182-5p inhibitor group when compared with control group. We found that the miRNA-182-5p is a promising therapeutic target in colitis treatment.

Inflammation response and oxidative stress are closely associated with the initiation and pathogenesis of colitis. The inflammation response is the eternal theme in colitis therapy. The inflammatory level is elevated in colitis and excessive generation of inflammatory cytokines causes colon injuries and contributes to the progress of colitis [43, 44]. Consistent with the previous literatures, both inflammatory factors and oxidative stress level were increased in model group [43, 44]. In this study, the inflammatory indicators including IL-21, IL-17 A, IL-6 were all declined in miRNA-182-5p inhibitor group when compared with model group. Our findings are largely consistent with a recent report in which miRNA-182-5p attenuates inflammation in cerebral ischemia-reperfusion injury[45]. These data suggested that inhibition of miRNA-182-5p is a promising strategy for inflammation control in colitis. It is known to all that oxidative stress is caused by disequilibrium between oxidative system and anti-oxidative system. In this research, the anti-oxidant indicator, SOD in model group was increased by miRNA-182-5p inhibitor. While the peroxidation product MDA, induced by DSS, was deceased by miRNA-182-5p inhibitor. Consistent with these findings, miR-182-5p inhibited oxidative stress and apoptosis triggered by oxidized low-density lipoprotein via targeting toll-like receptor 4[46].In addition, TGF-β1, which is generated by inflammatory and non-inflammatory cells, is vital immune reactions suppressor [47]. In this research, we found that the TGF-β1 level inhibited by DSS was elevated by miRNA-182-5p inhibitor. As discussed above, results support that downregulation of miRNA-182-5p has therapeutic effects in colitis.

Emerging evidence supports the notion that miRNA can complement and bind to target genes and directly cut mRNA, so as to regulate the level of target mRNA[48, 49]. The underlying mechanism for the role of miRNA-182-5p was further examined herein. We found that claudin-2 mRNA was targeted by miRNA-182-5p. Claudin-2 is one of the tight proteins which play pivotal roles in maintaining intestinal mucosal permeability. The tight junction proteins are essential in maintaining integrity of intestinal barrier. Dysfunction of intestinal barrier results in rectal bleeding and diarrhea, contributing to the onset and development of colitis [50]. In the present study, the decrease of claudin-2 was found in model group. After inhibition of miRNA-182-5p, claudin-2 was up-regulated evidently. This indicated that miRNA-182-5p functions in colitis via targeting claudin-2 and regulating claudin-2 expression.

The effects of claudin-2 overexpression were investigated in colitis to determine whether inhibition of miRNA-182-5p functions via upregulation of claudin-2. The pathological changes, colonic length, damage index in model group and TGF-β1 levels were all found to be improved by claudin-2 overexpression, which was consistent with the results in miRNA-182-5p inhibitor group. Such observation is in fact consistent with previous studies [51, 52]. Claudin-2 overexpression also exerted anti-inflammation and anti-oxidative stress effects. Collectively, all the effects of miRNA-182-5p inhibitor in colitis can be achieved via claudin-2 overexpression. Claudin-2 overexpression was also found in miRNA-182-5p inhibitor group, strongly elucidating that inhibition of miRNA-182-5p exerts protective effects in colitis via targeting and up-regulating claudin-2 expression.

## Conclusions

In the present study, miRNA-182-5p was overexpressed in colitis model and inhibition of miRNA-182-5p alleviated experimental ulcerative colitis induced by DSS via targeting claudin-2 *in vivo*. The effects included anti-inflammation, anti-oxidation, up-regulation of TGF-β1 and improvement of pathological damages. While the effects of miRNA-182-5p inhibition in colitis is still needed to be validated in clinic.
